# Ethical aspects of creating human–nonhuman chimeras capable of human gamete production and human pregnancy

**DOI:** 10.1007/s40592-015-0031-1

**Published:** 2015-10-12

**Authors:** César Palacios-González

**Affiliations:** Institute for Science Ethics and Innovation, The University of Manchester, Room 3.383, Stopford Building, Oxford Road, Manchester, M13 9PL UK

**Keywords:** Chimeras, Human gametes, Xenopregnancy, Human–nonhuman chimeras, Biotechnology, Part human

## Abstract

In this paper I explore some of the moral issues that could emerge from the creation of human–nonhuman chimeras (HNH-chimeras) capable of human gamete production and human pregnancy. First I explore whether there is a cogent argument against the creation of HNH-chimeras that could produce human gametes. I conclude that so far there is none, and that in fact there is at least one good moral reason for producing such types of creatures. Afterwards I explore some of the moral problems that could emerge from the fact that a HNH-chimera could become pregnant with a human conceptus. I focus on two sets of problems: problems that would arise by virtue of the fact that a human is gestated by a nonhuman creature, and problems that would emerge from the fact that such pregnancies could affect the health of the HNH-chimera.

## Introduction

In the second half of the twentieth century, interspecific chimeras went from the realm of mythology and literary studies to being created and studied by the faculties of life sciences around the world. Today, most human–nonhuman chimeras (henceforth HNH-chimeras) are used to investigate and model human biological functions and diseases that would be difficult to study in other settings (e.g. cell cultures or computer simulations).

Chimeras are formed by combining whole cells of genetically different organisms into a single functional organism. The UK Academy of Medical Sciences provides this definition:

Chimæras are formed by mixing together whole cells originating from different organisms. The new organism that results is made up of a “patchwork” of cells from the two different sources. Each cell of a chimæra contains genes from only one of the organisms from which it is made. (…) Primary chimæras are formed by mixing together two early embryos, or an early embryo with isolated embryonic cell types obtained from a different embryo or cultured stem cell line. The resulting chimæra has cells of different origins, in many tissues. Secondary chimæras are formed experimentally by transplanting (or grafting) cells or tissues into animals at later stages of development, including late fetal stages, post-natal or even adult animals. The donor cells are only present in a few tissues (The Academy of Medical Sciences 2011, pp. 18–19).

HNH-chimeras (in particular humanized mice) have been used in research into human autoimmunity, hematopoiesis, and cancer biology (Shultz et al. 2007). They may further serve in the development of vaccines against deadly diseases (Bhan et al. 2010; Davis and Stanley 2003; Sacci Jr. et al. 2006), and the development of human organs for transplantation (Rashid et al. 2014). It should be emphasised that the number and origin of the cells, and the timing of the mixing could produce very different outcomes in respect to the kinds of characteristics a chimera could possess (Greely et al. 2007; Karpowicz et al. 2005; The Academy of Medical Sciences 2011).

In most respects, the creation and use of HNH-chimeras for research purposes is not regarded as presenting additional ethical concerns alongside those related to the destruction of human embryos, animal ethics, and research ethics. In part, this has been the case because such entities have been predominantly constituted by nonhuman components, with only a few human cells. Nonetheless, there are three specific types of HNH-chimeras that have raised red flags among policymakers, researchers, and ethicists:

1. HNH-chimeras that could have brains predominantly constituted by human brain cells;
2. HNH-chimeras that could look human-like;
3. HNH-chimeras capable of human gamete production and human pregnancy.

Of these three cases, the one that has generated the most academic debate is the possible creation of HNH-chimeras with brains predominantly constituted by human brain cells (e.g. the neuron mouse, Greely et al. 2007). The other two cases have not been comprehensively explored; I presume that this is in part due to the fact that the ethics debate around the creation of HNH-chimeras is fairly recent. In fact, only Greely (2013) has explored some of the ethical issues regarding the creation of these three types of ‘sensitive’ cases. The aim of this paper is to further discuss the moral issues surrounding the creation of HNH-chimeras capable of human gamete production and human pregnancy.

At this point, someone could state that the ethics of creating HNH-chimeras capable of human gamete production and human pregnancy has been dealt with by three major advisory groups (the UK Academy of Medical Sciences, the German Ethics Council, and the US Committee on Guidelines for Human Embryonic Stem Cell Research), and that all of them have recommended either not creating HNH-chimeras that could produce human gametes or not letting HNH-chimeras breed. To such a claim I would reply that these three groups have *only* stated that such courses of action should not be taken; they have not elaborated on the ethical reasons that ground such prescriptive measures.

Next I present the recommendations of these groups. The Academy of Medical Sciences has stated that:

A very narrow range of experiments should not, for now, be licensed because they either lack compelling scientific justification or raise very strong ethical concerns. The list of such experiments should be kept under regular review by the proposed national expert body, but should at present include: (…) Breeding of animals that have, or may develop, human derived germ cells in their gonads, where this could lead to the production of human embryos or true hybrid embryos within an animal (The Academy of Medical Sciences 2011, p. 111).

The Committee on Guidelines for Human Embryonic Stem Cell Research has recommended that:

Embryonic Stem Cell Research Oversight (ESCRO) committees or their equivalents should divide research proposals into three categories [a, b and c] in setting limits on research and determining the requisite level of oversight: (…)

(c) Research that should not be permitted at this time. (…)

(iii) No animal into which hES cells have been introduced at any stage of development should be allowed to breed (National Academies of Science 2005, p. 58).

While the US and UK groups focused on the reproduction of HNH-chimeras, the German group focused on the creation of HNH-chimeras that could produce human gametes:

In addition to these limits [those of the German Embryo Protection Act, Sect. 7], the following additional prohibitions should be incorporated in the Act:

- prohibition of the transfer of animal embryos to humans;
- prohibition of the insertion of animal material into the human germline;
- prohibition of procedures potentially resulting in the formation of human egg or sperm cells in an animal (Deutscher Ethikrat 2011, p. 113).

With these recommendations in mind, I will first explore whether there is a cogent moral argument for not creating HNH-chimeras that could produce human gametes, and then what moral problems could emerge from the fact that a HNH-chimera could become pregnant with a human conceptus.

## Arguments against creating HNH-chimeras capable of human gamete production

In this section I explore whether there is a cogent argument that could ground the claim that the creation of HNH-chimeras capable of producing human gametes is morally problematic, as the German Ethics Council appears to think (Deutscher Ethikrat 2011).

Given that the German Ethics Council has not advanced a specific argument against the creation of HNH-chimeras that could produce human gametes, I will depart from a hypothetical strong normative claim, and I will then investigate whether there is an argument that could justify it. In doing so, I will try to build a strong case against the creation of this specific type of HNH-chimera that does not rely on an appeal to religious beliefs.

The normative claim is:

(A) It is morally wrong to create HNH-chimeras that could produce human gametes.

At first glance, there are two possible ways in which we could try to justify (A). The first points to the general immorality of creating *any* type of HNH-chimera, and the second to the specific value of human gametes. Let us examine the first one.

### The general wrongness of creating HNH-chimeras

An argument regarding the general wrongness of creating HNH-chimeras would claim something like this: creating *any* type of HNH-chimera is morally wrong. HNH-chimeras that could produce human gametes are a subset of the set HNH-chimeras. Therefore it is morally wrong to create HNH-chimeras that could produce human gametes.

While this argument might appear compelling, it fails because it rests on the soundness of the first premise, and this premise is false. There are several arguments that have been advanced in order to try to demonstrate its truth, and all of them have been found wanting: Robert and Baylis (2003) have examined and refuted the claim that crossing species boundaries is morally wrong; Robert and Baylis (2003) examined whether the creation of HNH-chimeras would produce a state of inexorable moral confusion that rendered their creation morally problematic (they did not take any stance on this issue), and later on Harris (2010), and Haber and Benham (2012) examined and refuted such a proposition; Karpowicz et al. (2004, 2005) and Cohen (2007) have examined and refuted the assertion that creating HNH-chimeras is morally wrong because they are unnatural or they transgress a moral taboo; Savulescu (2013) has examined and refuted the claim that HNH-chimeras would inherently threaten our humanity; and finally, DeGrazia (2007) and Palacios-González (2015) have refuted the charge that the creation of HNH-chimeras is morally problematic because it violates ‘human’ dignity.

At this point, one could argue that what has actually been concluded in most of the previous papers is that, *in principle*, the creation of nonperson-HNH-chimeras (that are predominantly nonhuman) is morally permissible, but that *the morality of creating HNH*-*chimeric*-*persons is still an unresolved moral issue*. It is worth noting that most discussions use a Lockean, or Lockean-like, account of personhood, where a person is “a thinking intelligent being, that has reason and reflection, and can consider itself as itself, the same thinking thing in different times and places” (Locke 1979, bk. II, Chap. 27, Sect. 9).

Let us grant for the sake of argument that creating HNH-chimeric-persons is morally problematic and should not be attempted. Now, even if we accept this claim, this does not entail a *principled point* against the creation of HNH-chimeras that could produce human gametes. Why not? Because human gametes can be produced by nonperson-HNH-chimeras. For example, we could create a mouse–human chimera that had mouse-proper mental capacities but that could produce human gametes.

### The value of human gametes

Given that a principled argument against creating *any* HNH-chimera has not been found so far, those who hold (A) could still try to defend it by advancing the argument that human gametes have certain worth that would be *debased* if they were to be generated within nonperson-HNH-chimeras. Commenting on the possibility of human gametes having certain worth, Greely has claimed that:

Unless one sacralizes human gametes, there is no reason to be any more concerned about their mere presence in a human/nonhuman chimera than there is to be concerned about the presence of liver cells, kidney cells, or tumor cells—and far less than for human neurons. (…) In the normal course of events, sperm and eggs are not cherished, protected, and treated with respect. In all humans’ lives, their gametes live and die, in the billions for men and in the hundreds for women, almost always futilely and never mourned or subject to “proper” or “respectful” disposal (Greely 2013, p. 684).

While most people would agree with Greely’s remark, it is important to highlight that the reasons he presents to show that human gametes do not have worth are problematic.^1^ In acknowledging the fact that people do not cherish, protect, and treat human gametes with respect, it does not necessarily follow that they possess no worth. People’s beliefs can match, or not, the type of worth an entity possesses. For example, people used to believe that normal cows had no moral worth. The fact that some believed such a thing did not make it true. Given this problem with Greely’s remark, it is better to present a different argument.

There are two types of worth that human gametes could have: they could have intrinsic worth or they could have instrumental worth. Defending the claim that human gametes have intrinsic worth—understanding intrinsic worth as possessing moral status—is a nonstarter. A being possesses moral status when “*in its own right and for its own sake, it can give us reason to do things such as not destroy it or help it* [emphasis in the original]” (Kamm 2007, p. 229). Human gametes, in their own right and *for their own sake*, cannot give us reasons not to destroy them or to help them, since they do not have interests. Even those who contend that the human embryo is a person (or potential person)^2^ admit this (Gómez-Lobo 2004). Now, even if we were to accept the *outlandish* claim that human gametes have intrinsic worth, it is difficult to see why it would be debased by virtue of being created within a nonperson-HNH-chimera. In principle, an entity that possesses intrinsic worth possesses it independently of external circumstances, such as the place where it was created.

The second option is for human gametes to have instrumental value. Given that instrumental value is dependent on the making or completion of a task, we can indeed assert that human gametes can possess instrumental value. For example, when someone resorts to IVF, eggs and sperm become valuable as means to achieve reproduction.

Now, even if human gametes have only instrumental value, someone could press the argument by claiming that their instrumental value would *always* be debased by being generated within a nonperson-HNH-chimera, and therefore that we should not create chimeras that could produce them. The problem with this claim is twofold. First, we could try to achieve certain specific goals with chimeric-generated human gametes that we would not want to achieve with human-generated human gametes, and thus it is in relation to their usefulness for achieving such goals that their instrumental value should be measured.

Second, even if the instrumental value of chimeric-generated human gametes were measured against the goals that human-generated human gametes can achieve, this does not *necessarily* entail that chimeric-generated human gametes would have less instrumental value. Why not? Because if the value of chimeric-generated human gametes is related to achieving certain goals, within certain standards, then as long as they achieve these goals, it is simply not true that their instrumental value would be debased. This means that if the final result (reproduction or research) is not affected by the circumstances of their generation (inside a nonperson-HNH-chimera), then both sets of human gametes (human-generated and chimeric-generated) could have the same instrumental value.

### Intuitive moral responses to HNH-chimeras capable of human gamete production

A further option is available to those who hold that we should not create chimeric entities that could generate human gametes: to appeal to our intuitive moral responses. Within the literature on the ethics of creating HNH-chimeras, we find an example of this in Streiffer’s commentary to the Robert and Baylis (2003) paper ‘Crossing Species Boundaries’. There he states that “[e]ven opponents of the yuck factor [an intuitive negative moral response] must concede that, sometimes, we know that an action is wrong merely on the basis of our reaction to it, even if we cannot satisfactorily justify that reaction” (Streiffer 2003, p. 38).

The first problem with this strategy when using it to ground a normative claim is that different people have different intuitive moral responses regarding the same subject. This means that we tend to end up with many contradictory intuitions that try to establish different normative claims. For example, some might intuit that creating nonperson-HNH-chimeras that could produce human gametes is morally unproblematic, while others might intuit the opposite.

It is true that our intuitive moral reactions to certain states of affairs are strong, but it is important to qualify what role those intuitions should play in our moral life. In recent work, Jonathan Glover has advanced the idea that such intuitive moral responses can have at least two functions: as excluders or as early warning systems. He goes on to assert that we must have a great deal of confidence in our intuitive moral responses for them to be used as excluders [i.e. “a sign that a morally impassible barrier has been reached” (Glover 2015, p. 38)]. This is so because the intuitive moral response is taken as a final evaluation regarding the subject that is being examined. For example, think of someone whose intuitive moral response to interracial marriage was deeply negative, and that she used such a response as an excluder that settled the matter. It is true that later on such a person can assess, from an ethical theory, whether the situation that prompted the intuitive negative moral response is, in fact, immoral. The problem here is that if the person strongly believes that her intuitive moral responses are *always* right, she has no reason to doubt them or to thoroughly examine them.

Even if this person does not believe that her intuitions are morally infallible, she can still use them as excluders by acknowledging that, at certain times, we cannot satisfactorily justify our reactions, just as Streiffer asserts. For example, the above-mentioned woman could do exactly this if she were to find no good argument to support her intuitive moral response to interracial marriage. Settling for our moral intuitions, when no rational justification can be provided, seems morally risky, given the long history of human intuitions in support of deeply immoral practises (e.g. slavery).

An alternative approach to using our intuitive moral responses as excluders is to use them as an early warning system. When we use them in this fashion, we assume that they are first responses and are open to rational criticism and discussion. Glover divides our intuitive moral responses into three types: ‘trained moral nose’, ‘strange smell’, and ‘human responses’. The ‘trained moral nose’ responds to situations in accordance with a set of relevant moral beliefs. For example, when someone tries to disguise a religious argument as a secular argument, those with a trained moral nose will notice a ‘religious smell’. The trained moral nose relies little on intuition, given that arguments can promptly be provided, and thus we are no longer in need of the intuitive moral nose. The second source of intuitive moral responses are ‘strange smells’. These strange smells appear in unfamiliar situations that might threaten previously engrained category divisions. For example, when IVF first appeared, many felt that it had a ‘strange smell’—a smell that originated from the disruption of entrenched reproductive categories. The last moral nose is the ‘human response’. This nose is activated when we feel sympathy for others (people or certain nonhuman animals) that suffer or are denied respect. An important caveat regarding this one is that we can override it if there are sufficiently strong motives for doing so (Glover 2015, pp. 33–43).

If we regard our intuitive moral responses to the creation of nonperson-HNH-chimeras that could produce human gametes as an early warning system, we can then examine which of the three alarms it sets off. In the first part of Sect. 2.1, I have shown that those with a ‘trained moral nose’ have so far failed to show that the creation of nonperson-HNH-chimeras capable of producing human gametes is immoral. Those who hold that creating such chimeras produces a ‘strange smell’ could justify this by asserting that the creation of human gametes within chimeras is an unfamiliar situation that modifies a previously engrained category division (i.e. only humans produce human gametes). It is important to realise that people who detect this ‘strange smell’ do not necessarily need to hold that the creation of such chimeras is immoral. I am inclined to think that the ‘strange smell’ hypothesis is behind most intuitive moral responses to the creation of chimeras that could produce human gametes. Finally, some people can have a ‘human response’ when confronted with the creation of such chimeras. They would claim that using them for biomedical research or reproductive purposes is immoral, given that they are not properly respected. It is true that chimeras’ moral status would require from us certain things, but it is also true that if strong reasons are presented, these can trump the chimeras’ interests. What should we make of this discussion of our intuitive moral responses? That it is better to understand them as an early warning system, and that in the case of creating HNH-chimeras capable of human gamete production, they appear to be of either the ‘strange smell’ or the ‘human response’ kind.

At this point we can conclude that, so far, it has not been possible to construe a *principled* argument that grounds the hypothetical strong normative claim (A). At best, we could present particular arguments against using chimeric-generated human gametes for certain specific ends (if they were not good enough for attaining those ends).

## Arguments in favour of creating HNH-chimeras capable of human gamete production

Three arguments can be advanced in favour of creating nonperson-HNH-chimeras capable of human gamete production: the saving persons argument, the unburdening women argument, and the restoration of fertility argument. Of these three, only the first seems strong enough to justify the creation and posterior destruction of such chimeras. The second and third arguments could justify the extraction of such gametes (which would imply a certain degree of harm), but it is not clear that they could justify the destruction of the chimeras.

### The saving persons argument

Let us examine the saving persons argument. By producing chimeras capable of producing human gametes, we could reduce the shortage of human eggs. This shortage is relevant because it slows, and in certain cases stops, valuable medical research that could save lives (Maher 2008). For example, two areas that would benefit from a surplus of human eggs are regenerative medicine and embryonic stem cell research. Now, with regard to obtaining the gametes, there are two options: the chimeras could be terminated after the extraction, or they could not. In either of these two scenarios, the extraction of eggs is morally permissible if killing sentient nonpersons in order to save persons’ lives is morally permissible.

It should be noted that the strength of this argument also depends on two further facts. The first is that iPS cells cannot replace hESCs, and that in vitro gametogenesis (from which human embryos would be produced and then embryonic stem cells derived) cannot replace hESCs. If the contrary were the case, then harming or killing such chimeras would be immoral. The second requisite is that the harms that a chimera would endure are proportionally less than those that a human person would have to endure by donating her eggs. If the harms to the chimeras were equal to or larger than those experienced by a human person, justifying the morality of such practices would be more difficult.

### The unburdening women argument

The second argument that we could put forward for favouring the production of these chimeras is that of unburdening women. By means of creating nonperson-HNH-chimeras capable of producing human gametes, we would reduce the number of women subject to the risks that donating eggs for research purposes entails. This second reason would extend to egg extraction, or donation, for reproductive purposes if chimeric-generated gametes could, and would, be used for such purposes. Among the medical risks of egg donation are mild, moderate or severe ovarian hyperstimulation syndrome, ovarian torsion, infection, rupture of ovarian cyst, and intra-abdominal bleeding. Some of these risks are moderate, while others can be life threatening (Maxwell et al. 2008).

Three things should be noted about this second argument. First, it can be regarded not as an independent argument but as a positive externality of the first argument. Second, if it is construed as an independent argument, we must further show when such acts are, in fact, morally permissible. Why? Because while it seems permissible to harm the chimera in order to unburden women, it is not obvious that the possible harms to the human person are reason enough for killing the nonperson-HNH-chimera.^3^ I will not expand on this point, because here I am taking this second argument not as an argument *per se* but as a positive externality of the first argument.

The third thing to note is that this second argument could in certain scenarios be used as a counterargument to the creation of such human gamete-producing chimeras.^4^ If the quality and usefulness of such chimeric-generated human eggs were inferior to those produced by women, but not to the point of being useless, they could simply be used as an intermediate step in experiments that in the end would require women’s eggs anyway. Suppose that scientists are trying to achieve X and that they employ chimeric-generated gametes as a way to do so. They realise that such gametes are only partially appropriate for the research. If this were the case, they could use chimeric gametes to get closer to X, and then at some point they would switch to using women’s eggs. The creation of nonperson-HNH-chimeras that could produce human gametes would, in these circumstances, end up promoting egg donation, with all the dangers that it implies.

Three things should be noted regarding this possible counterargument. The first is that it is dependent on the attainment of certain empirical facts. The only way we have of knowing whether chimeric-generated human gametes could be equivalent (in terms of research or reproductive potential) to human-generated human gametes is to actually develop and test them (while constantly trying to refine the ways in which they are generated). The second point is that chimeric-generated human gametes could be used as an alternative to human-donated eggs while other methods for generating human gametes are perfected—for example, the mass production of in vitro-generated gametes from induced pluripotent stem cells (Palacios-González et al. 2014). The third point is that even if researchers eventually need additional quantities of human-generated human eggs, this is not necessarily bad overall, despite the possible dangers. The harms of egg donation in such circumstances should be weighed against the good that could be done—supposing, of course, that such donations are made with proper informed consent, and not under duress or undue influence.

### The restoration of fertility argument

The third argument, the restoration of fertility, claims that restoring human fertility is of such importance that we should create and use nonperson-HNH-chimeras that could produce human gametes for this end. This might be an option, for example, when patients become infertile due to medical treatments (such as cancer treatment) or trauma. In fact, research into this area has already started. Scientists have *xenografted* human reproductive tissues into young Swiss nude and SCID-NOD [non-obese diabetic/severe combined immunodeficient] mice in order to study how to preserve the fertility of patients enduring gonadotoxic cancer treatments (Arregui and Dobrinski 2014; Geens et al. 2006; Kim et al. 2005; Weissman et al. 1999; Wyns et al. 2008). Now, the merit of this third argument depends on whether the strength of the reproductive interests of such humans would trump over the interests of the nonperson-HNH-chimeras. While the case where a chimera would be harmed, but not killed, by the retrieval of the gametes seems morally permissible, it is not clear to me that the cases where the chimera would be killed are equally so.

To sum up, three different arguments can be advanced for creating and using nonperson-HNH-chimeras capable of producing human gametes. Of these three arguments, the first (the saving persons argument) is the strongest, given that it morally permits harming and killing such chimeras. The other two arguments (the unburdening women argument and the restoration of fertility argument) seem to permit harming the chimeras, but it is not clear that they would allow for their killing (although this is open to further investigation).

At this point we could offer a different explanation of the German Ethics Council’s stance. Instead of assuming that their recommendation is grounded on a moral claim about the general wrongness of creating HNH-chimeras that could produce human gametes (or the debasement of the value of human gametes), we could posit that their claim is a practical recommendation in order to avoid human or hybrid pregnancies within HNH-chimeras. As Greely has noted:

The worry [about creating HNH-chimeras that could produce human gametes] *must* be not about the gametes in themselves, but about the possibility that the gametes will be effectively used—that human sperm or eggs in chimeras will fertilize or be fertilized (Greely 2013, p. 684).

Now I will turn to some of the ethical problems that could emerge from the reproduction of nonperson-HNH-chimeras that produce human gametes.

## Human pregnancies within nonperson-HNH-chimeras

Depending on the biological makeup of the nonperson-HNH-chimeras, the following pregnancies are *theoretically* possible: (i) nonhuman animal pregnancy, (ii) hybrid pregnancy (e.g. a human egg is fertilized by a nonhuman sperm), or (iii) human pregnancy. In this section I will explore the third possibility, and by this I mean the moral quandaries that could arise from the fact that nonperson-HNH-chimeras could get pregnant with human conceptus.^5^

There are three possible ways in which human chimeric pregnancies might occur: (i) sexual reproduction, (ii) artificial insemination, or (iii) IVF and embryo transfer. It is important to bear in mind that only sexual reproduction (if the biological conditions are met) can be achieved without human intervention. This means that nonperson-HNH-chimeras’ human pregnancies could be either intentionally sought or could happen accidentally.

In order to *avoid* accidental human pregnancies, Greely has proposed (i) creating HNH-chimeras of only one sex, (ii) using HNH-chimeras that are reproductively immature, and euthanizing them before they reach reproductive maturity, (iii) sterilizing them, (iv) euthanizing them if they get pregnant, or (v) physically segregating them by sex (Greely 2013, p. 686).

A human pregnancy within a nonperson-HNH-chimera could raise moral concerns for two sets of reasons:

(B) It could be morally problematic because of what could happen to the human conceptus.
(C) It could be morally problematic because of what could happen to the nonperson-HNH-chimeras during pregnancy and labour.

Although here reasons (B) and (C) can be neatly divided, in actual pregnancies they could overlap, adding complexity to the moral evaluation of such cases. Let us start by examining (B). There are three things that could happen to any embryo produced within a nonperson-HNH-chimera that could be regarded as morally problematic.

1. It could die in utero due to intrinsic biological circumstances.
2. It could be terminated by human action.
3. It could develop well and long enough to be able to survive in the extrauterine environment.

Let us examine each of these scenarios.

### It could die in utero due to intrinsic biological circumstances (B.1)

Before discussing (B.1) it is important to realise that almost all reproductive scenarios involving HNH-chimeras will probably fall under this heading. Why? Because HNH-chimeras that are currently used for research are biologically incapable of carrying to term any human pregnancy (e.g. mice–human or pig–human chimeras). As Greely rightly says, “[d]epending on the nonhuman component of the chimera, a 'human' pregnancy could be disastrous; no human fetus could develop successfully inside a mouse or a rat” (Greely 2013, p. 685).

There are three scenarios that fall under (B.1) that could be regarded as morally problematic: (B.1.1) the human embryo could fail to implant in the body of the nonperson-HNH-chimera and therefore die; (B.1.2) it could implant, start to develop, and die without any detrimental effect to the health of the nonperson-HNH-chimera; or (B.1.3) it could die after implantation, and such event could have a detrimental effect on the health of the nonperson-HNH-chimera.^6^

One of the reasons why *natural* human embryo loss (i.e. that which depends on the intrinsic characteristics of the embryo or the intrinsic biological traits of the woman) is not regarded as a morally problematic event in itself is because the loss is not intentionally sought, and therefore no one is blameworthy. If this is the case for human-occurring human pregnancies, all else being equal, the same reasoning should then apply to cases of *natural* embryo loss in chimeric pregnancies. Why? Because if moral blameworthiness rests on the fact that the loss is intentionally sought, then as long as it is not, there appears to be no moral problem in this regard.

One could argue that naturally occurring human embryo loss is morally problematic in cases of nonperson-HNH-chimera pregnancies. Why? Because scientists are in fact intentionally *creating*, or facilitating the creation of, human embryos for research purposes that would be ‘destroyed’ by means of ‘placing’ them in an environment where they cannot survive. Thus, even when the destruction of the embryo is not *intentional* per se, it is in fact foreseeable, and it is morally wrong not to stop a foreseeable bad outcome that can indeed be stopped.^7^

There are two problems for those who hold this position. The first is that they would necessarily be committed to the claim that women who knew that they cannot carry a pregnancy to term because of certain medical conditions would be doing something morally wrong when placing themselves in a position where they could get pregnant. This strike us as evidently false.

The second problem is that this counterargument depends on the human embryo possessing moral worth—worth that is far from proven. Why is this the case? Because if there is something morally wrong with scientists participating in, or causing, a human pregnancy within a nonperson-HNH-chimera that will not come to term, then it must be, in part, because something valuable would be lost. In this case, the thing considered to be of value is the embryo’s life. However, human embryos lack the morally relevant properties that would make their intentional, or foreseeable, destruction morally wrong. Even more so, the fact that they possess such properties in a potential state does not, in actuality, confer them moral worth. What this means in practical terms is that their destruction, intentional or accidental, is of no moral consequence^8^ (Devolder 2005; Devolder and Harris 2007; Harris 1985).

Following a similar line MacKellar and Jones have advanced a ‘confusion’ argument against causing or allowing *any* nonperson-HNH-chimera human pregnancies. For them “[t]here is indeed a risk that human embryos and human foetuses could be considered in the same manner as those of animals (with no special protection being granted) if they were to be found in an animal. As a result, this may further undermine the conferring of any respect and dignity on human embryos and foetuses” (MacKellar and Jones 2012, p. 81).

The force of this argument is not as strong as these authors believe: this concern applies neither to those who think that the human embryo possesses an intrinsic worth comparable to that of a normal adult human being, nor to those who think that the human embryo does not possess intrinsic moral worth. It does not apply to the former group because they, *in principle*, accept the assertion that regardless of where such embryos are located, they possess intrinsic moral worth.^9^ It does not apply to the latter group because they, also *in principle*, reject the intrinsic moral worth of the embryo, regardless of where the embryo is. Thus the risk of considering the human embryo as a thing that it is not (i.e. a nonhuman animal embryo) should be understood as a risk of *biological* confusion and not as a risk of *moral* confusion. Additionally, if the human embryo does not possess intrinsic moral value, then the distinction between embryos with ‘dignity’ and embryos without ‘dignity’ does not hold, given that *all* of them are dignity-less embryos. At this point we can conclude that cases (B.1.1), (B.1.2), and (B.1.3) are morally unproblematic from *a human embryo loss perspective*.

Now, causing or allowing pregnancies of the type (B.1.1) and (B.1.2) could be regarded as morally unproblematic, because on the one hand, human embryos do not have intrinsic moral worth, and on the other hand, the health of the nonperson-HNH-chimeras would not be jeopardized by the pregnancy. While this might be the case, we should not overlook the fact that all of the chimera’s interests, not only its health, should be taken into account when assessing the different courses of action. Case (B.1.3) is, in an important respect, different from (B.1.1) and (B.1.2). The difference is grounded on the fact that from the start, we know that such pregnancies will have a negative toll on the chimera’s health. This being the case, scientists should present strong moral reasons either for causing such human pregnancies or for not promptly terminating them.

The practical implications of this section are thus: there would be nothing morally wrong with allowing or causing human pregnancies to occur if they are of type (B.1.1) or (B.1.2), and if the human pregnancy would have a detrimental health effect on the nonperson-HNH-chimera, as in case (B.1.3), we must then provide strong reasons for causing or letting such pregnancies continue.

### It could be terminated by human action (B.2)

Now I will explore (B.2). Here, I do not address whether it is permissible to terminate a human pregnancy, but what moral reasons there could be to terminate a human pregnancy that occurs within a nonperson-HNH-chimera.^10^ There are at least six reasons that could be advanced to show that we are morally required to terminate a nonperson-HNH-chimera human pregnancy: (B.2.1) because of the ill effects such pregnancies would have on the health of the nonperson-HNH-chimeras; (B.2.2) because the human will nonetheless eventually die in utero; (B.2.3) because the child born would have a wrongful life; (B.2.4) because, if carried to term, such a human is likely to have major health problems; (B.2.5) because such pregnancies would be morally repugnant; and (B.2.6) because any child born this way would be severally discriminated against. Before examining these cases, it is important bear in mind that cases (B.2.3), (B.2.4), (B.2.5), and (B.2.6) would necessarily require that the human conceptus is viable, so for the sake of argument, I will treat these four scenarios as feasible.

In a research context, case (B.2.1), along with (B.1.3), would morally require the termination of such humans if (i) the scientific gains from such pregnancies do not outweigh the harms that the nonperson-HNH-chimeras would suffer from it, or (ii) there are no scientific gains from such pregnancies. This is grounded on the fact that, whereas human embryos do not possess intrinsic value (as previously stated), nonperson-HNH-chimeras would be sentient^11^ (which confers upon them moral worth), and their interests should be weighed against possible scientific gains.

On the other hand, if there were an instance (B.2.1) where there would be detrimental health effects for the nonperson-HNH-chimera but the human would be viable, and (i) the group in charge decided not to terminate the pregnancy, regardless of the law and the health consequence to the nonperson-HNH-chimera, or (ii) the pregnancy somehow remained undetected until the conceptus was able to survive in an extrauterine environment, then those in charge of the chimera would have to tackle the *really substantial* practicalities that a human being born from such entity would entail. By this I do not mean the certain media frenzy, but what should be done with a child born in these circumstances. I think that even in this farfetched scenario, two things should be obvious: (i) in terms of child protection, such cases should be treated *on a par* with cases of fully incompetent family-less women who are pregnant, and (ii) regardless of her *gestational origins*, such child would be entitled to the same treatment as any child born to a woman, given that her moral status would be the same as that of any other human child. It should be noted that while most conservatives would much rather reject the idea of a nonperson-HNH-chimera getting pregnant with a human conceptus, as soon as it got pregnant, they would necessarily be committed to the defence of such life, since for them, a human embryo has the same moral status as that of a normal adult human.

If the human conceptus will eventually die in utero, case (B.2.2), either by legal requirement or by natural causes, we would be morally required to abort it as soon as possible when there were no scientific gains to expect from its development, and when its existence negatively affects the health (or other interests) of the nonperson-HNH-chimera (just as in cases (B.2.1) and (B.1.3)). The abortion should take place early on, given that late-term abortion could have a deeper negative impact on the chimera’s health.^12^ Finally, as stated above, if there are scientific reasons for letting the pregnancy carry on, then such reasons should be weighed against the interests of the nonperson-HNH-chimera.

Of all the cases, I think that (B.2.3) might be the easiest to deal with, because morality requires that either wrongful lives (i.e. lives of such overall poor quality that they would have been better to never have been) should not be brought to birth (when endorsing an impersonal account of morality) or, if so, they should be terminated as soon as they start to suffer (when endorsing a person-affecting account of morality).^13^ In practical terms this means that if we knew that the developing human would have a wrongful life, we would then be morally compelled to terminate her. And, just as stated above, if there were possible scientific gains that could be obtained from such pregnancies, that override the health interests of the nonperson-HNH-chimera, then the developing human should be left to develop only for as long as her life is non-wrongful.

Now let us suppose that a nonperson-HNH-chimera is pregnant with a human, and that all the scientific data gathered pointed to the conclusion that even when the developing human has major health problems, she is viable and would have a life worth living. What should we do in such cases? Although, as Hank Greely rightly recognises, “[m]any countries, including the United States, allow abortion of fully human fetuses for broad reasons, and others allow abortion for reasons of fetal health” (Greely 2013, p. 685), I think that (B.2.4) cases do not straightforwardly require the termination of such conceptus. As the non-identity problem shows us, as long as such human life is worth living, we would not make her worse off by not aborting her (Parfit 1984, Chap. 4), because the only other option for her would be to never have been.^14^ In point of fact (and as stated previously), we should treat (B.2.4) in the same way as we would treat pregnancy cases of family-less fully incompetent women (that had the same capacities as the nonperson-HNH-chimera) who are pregnant with a developing human that has severe health problems. I think moral consistency requires this action and nothing less. I also maintain that if someone recommended treating those cases in different ways, because in one instance the *pregnant creature* is fully human and in the other the creature is a chimera, such call for action would clearly be speciesist. Now, if we are not morally required to terminate those humans that have severe health problems and are gestated by family-less fully incompetent women (even if doing so is not morally problematic), we are then also not morally required to terminate the pregnancy of a nonperson-HNH-chimera that gestates a human with severe health problems (it should be added that we are required to take into consideration the interests of the pregnant creature when deciding the course of action, and that this might yield different results). If this ever happened, just as was discussed in (B.2.1), those in charge of the chimera would be required to take appropriate measures for the child to be properly taken care of.

Case (B.2.5) should be classified as no more than a ‘yuck factor’ instance, as explained in (2.3). The fact that these pregnancies might prompt strong intuitive moral responses is not reason enough for terminating them. Suppose that a child with a life worth living could be born from a human-chimpanzee chimera. If this were the case, then what could be regarded as repugnant? Someone might claim that such pregnancies are repugnant because they are unnatural or because they cross the species barrier. This objector to nonperson-HNH-chimera human pregnancies has the burden of proof to show that such arguments are different from those that have already been levelled against the creation of any type of HNH-chimera (see Sect. 2.1), and that have been found wanting. I think that in order to be significant for debate, any claim stating that such pregnancies are morally repugnant must be backed up by *explicit* rational arguments.

Finally, someone could claim that the main moral reason we have for terminating such pregnancies is that any child born of them would be harshly discriminated against, case (B.2.6). Many might think that this is the most compelling argument against nonperson-HNH-chimeras giving birth to a human child, but I consider this point to be terribly misleading. As I have previously said, a human child born of a nonperson-HNH-chimera would have the same moral status as any other human child born to a woman. This means that society (scientists included) should treat her accordingly to her moral status, and this entails that she is not discriminated against on the basis of who bore her. The fact that such a child would be born of a *different* type of entity should in no way be detrimental to the child’s flourishing and opportunities. If we think that discrimination against a child because of her origins is wrong (regardless of what these origins are), then (B.2.6) should not be taken seriously as an argument for terminating such pregnancies. Think of the reaction if a racially mixed couple were told that they should not have a child because the child would be the object of discrimination due to her ‘strange’ origins. Such advice is morally ill informed, and in such situations the proposed prescriptive measure is immoral and thus does not warrant enactment.

From this section we can conclude that unless the life of the developing human would be a wrongful one, we are *notmorally required* to terminate nonperson-HNH-chimera human pregnancies in *all* cases.

### It could develop well and long enough to be able to survive in the extrauterine environment (B.3)

What moral problems could there be if a human is successfully born from a nonperson-HNH-chimera? Although I think that this could be perceived by some as the most outrageous scenario, it is actually not especially problematic. This is because, as I stated earlier, a child born from a nonperson-HNH-chimera would have the same moral status as any other human child. At this point, someone could claim that it is unreal to think that such a child would be treated adequately, given that she would be born either as part of an experimental project or as an accident. Such person could also claim that being born under these circumstances would mean that this child would never be accorded moral status, and that this is reason enough for not letting nonperson-HNH-chimera human pregnancies happen.

My response to this claim is twofold. First, it does not follow from the fact that a future child could be mistreated (either intentionally or accidentally) that the only adequate response is to prevent her existence (once again, think of the case of the racially mixed couple). It is true that in the history of science, there are cases where scientists have failed to respect the moral status of their research subjects (for example, the Tuskegee syphilis experiment), so in order to tackle this possibility of mistreatment, scientific governing organisations should enact clear guidelines addressing how to proceed if these types of cases ever were to occur, on top of relying on the morality of researchers.

Second, it is important to realise that it would be very difficult to claim that a child that is generated by human gametes, produced by HNH-chimeras, and then brought to term by a HNH-chimera, has been made worse off^15^: it is highly likely that if these generative specific acts had not happened she would not have existed. We need to remember that a particular child’s existence is necessarily tied to the fusion of the specific gametes that produced her. This means that if she was not created at the specific time, under the specific circumstances that epigenetically affected her, and in the specific way in which she was created, then the most probable alternative is that either no child would have been created or a non-identical child would have been created. This means that as long as the child’s life is worth living, the type of generative action that brings her into existence does not make her worse off.^16^

Given that a child born from a nonperson-HNH-chimera would have the same moral status as any other child, the supposed moral problems generated are, in fact, not specific to chimera gestation, but are general problems about population ethics and child protection that we confront on a regular basis.

### Such pregnancies could be morally problematic because of what could happen to the nonperson-HNH-chimeras during the pregnancy or labour (C)

Another line of argument against allowing nonperson-HNH-chimeras to become pregnant with humans is that (i) we do not know what the consequences for the health of the nonperson-HNH-chimeras could be, and (ii) given that nonperson-HNH-chimeras do not lose anything by not becoming pregnant, we should not allow pregnancy to occur.

I will start with point (ii). It is true that the nonperson-HNH-chimera would not lose anything by not becoming pregnant with a human, and this is indeed an argument in favour of not allowing them to become so (just as we do not usually encourage fully incompetent women to get pregnant).^17^ On the one hand, if there is nothing that we could scientifically gain from such human pregnancies, then in order to not affect the chimera’s health (or other interests), we should prevent such cases from happening by following one of Greely’s recommendations. On the other hand, if there are scientific gains that could be obtained from allowing a human pregnancy (noting that the duration of the pregnancy would depend on current legislation), we might be losing something important if such pregnancies did not occur. This means that, depending on the scientific scenario, there might be moral reasons to either let such human pregnancies happen or not to let such pregnancies happen. For example, there is at least one theoretical possibility, whose morality is open to further elucidation, for why scientists might want to do this: if nonperson-HNH-chimeras could be used to create human organs for transplantation, then at a certain point they might try to create human female reproductive organs within a nonperson-HNH-chimera. If this were ever to be the case, scientists could be tempted to explore the functionality of such uteri by transplanting a human embryo into it and observing what happens.

The argument against letting nonperson-HNH-chimeras become pregnant with a human conceptus, which hinges upon the fact that we do not know what the health consequences for the chimeric entities could be, is similar to that explored in cases (B.2.1) and (B.1.3). Just as in those cases, the answer here is that we could be morally compelled to carry out such pregnancies, even against the health interests of the chimeric entity, when the scientific gains that would be obtained from the pregnancy outweigh the ill health effects on the nonperson-HNH-chimera. It is true that this threshold is high, but this does not imply that there are no circumstances in which it could be met.

## Final remarks

This work is divided into two broad parts. In the first part, I have tried to show that, so far, there is no good argument against the production of nonperson-HNH-chimeras that could produce human gametes. I have also advanced that there is at least one good moral reason for creating nonperson chimeras capable of producing human gametes: there is a shortage of human eggs for research. In the second part, I explored some of the ethical aspects that could be encountered if a nonperson-HNH-chimera were pregnant with a human. The moral problems that arise from such pregnancies fall into two categories. First, there are the moral problems that we would encounter if we fixed our sight on the product of such pregnancies. Second, there are the moral problems that would emerge from the fact that there is no previously gathered safety data from these pregnancies, and therefore no data on how the interests of the nonperson-HNH-chimeras could be affected. For both sets of problems, I have tried to provide some answers that hinge upon the moral status of the embryo and how moral consistency would require us to act in cases where a nonperson-HNH-chimera became pregnant with a human. Even when I have not given definitive answers, or provided a complete road map of the moral problems that could be present in such pregnancy cases, I think that this first mapping of the moral landscape is important insofar as it is a topic that has been neglected in the bioethical literature, but that could be technologically possible in the not-far-distant future.

## Footnotes

1 I would like to thank an anonymous reviewer for bringing this point to my attention.

2 A way of attacking one version of the potentiality argument (a human embryo has the moral worth of a normal adult human being because it has the potentiality to become one) is to point out that the unfertilized egg might also possess the potential to become a person. The unfertilized egg would possess the same moral worth as a normal adult human being by virtue of the possibility of technology-aided parthenogenesis (Devolder 2015; Devolder and Harris 2007). A possible defence against this attack is to further distinguish between the potentiality of a human embryo and that of a parthenote (Austriaco 2011).

3 An important caveat must be mentioned: this objection would not necessarily apply in all cases, because we could create HNH-chimeras that could produce human gametes and at the same time lack moral status. For example, we could try to create anencephalic pig-human chimeras that could produce human gametes.

4 Here I follow the argument advanced by Baylis (2008) when critiquing the creation of cytoplasmic hybrid embryos (such embryos are produced when human nuclear DNA is inserted into the enucleated egg of a nonhuman animal).

5 While I will not discuss the issues surrounding the reproduction of HNH-chimeric-persons here, it is worth pointing out what Cobbe has clearly stated: “[I]f a chimera with apparently human mental faculties should be either kept in isolation or unable to reproduce with similar creatures because of enforced prohibitions against breeding, this entails automatic denial of what many would otherwise see as fundamental rights in humans” (Cobbe 2011, p. 142).

6 Even when case (B.1.3) is part of the broader category of how human pregnancies could affect the interests of nonperson-HNH-chimeras, I will restrict my discussion to how the chimera’s health could be affected and the moral implications of this.

7 This objection does not apply to those cases where the process by which the embryo is formed makes it unviable.

8 For a discussion about how the legal permissibility of abortion would support a liberal policy on human embryonic stem cell research that involved the creation of HNH-chimeras, see Streiffer (2010).

9 I would like to thank an anonymous reviewer for bringing this point to my attention.

10 I take for granted that there is no further need to develop the arguments in favour of the legal and ethical permissibility of some abortions.

11 It could be the case that through further genetic engineering, nonperson-HNH-chimeras could be created to be non-sentient, see note 5.

12 I would like to thank an anonymous reviewer for bringing this point to my attention.

13 For a recent discussion on the moral permissibility of creating wrongful lives, see Smajdor (2014) and Williams and Harris (2014).

14 The fact that this human is not made worse off does not imply that under a non-comparative account of harm she could not be regarded as being harmed, nor that there could not be impersonal reasons for not carrying to term such pregnancies.

15 As discussed in (B.2.3) the only case in which a child could be made worse off by her existence is if she had a wrongful life.

16 Two questions are left open. The first is whether the numerical identity of an individual would be affected if she were either gestated by a HNH-chimera or a woman. The second is whether prenatal harm would ensue depending on whether one or the other gestates. For a discussion about possible persons and the problem of prenatal harm see Williams (2013).

17 I would like to thank an anonymous reviewer for bringing this point to my attention.
